# Correlates of Male Circumcision in Eastern and Southern African Countries: Establishing a Baseline Prior to VMMC Scale-Up

**DOI:** 10.1371/journal.pone.0100775

**Published:** 2014-06-23

**Authors:** Khai Hoan Tram, Jane T. Bertrand

**Affiliations:** 1 Stanford University School of Medicine, Stanford, California, United States of America; 2 Tulane University School of Public Health and Tropical Medicine, Department of Global Health Systems and Development, New Orleans, Louisiana, United States of America; University of the Stellenbosch, South Africa

## Abstract

**Background:**

Despite the importance of male circumcision (MC) prevalence to HIV prevention efforts in Eastern and Southern Africa, there has been no systematic analysis on the correlates of male circumcision. This analysis identifies correlates of MC in 12 countries in the region with available data.

**Methods:**

Data from the male questionnaire of DHS surveys collected between 2006–2011 in Ethiopia, Kenya, Lesotho, Malawi, Mozambique, Namibia, Rwanda, Swaziland, Tanzania, Uganda, Zambia, and Zimbabwe were analyzed. The dependent variable was self-reported male circumcision status. Independent variables included age, education, wealth quintile, place of residence, ethnicity, religion and region. Bivariate and multivariate analyses were conducted separately for each country.

**Results:**

MC prevalence ranged from 8.2 percent in Swaziland to 92.2 percent in Ethiopia. Bivariate analyses showed a consistent positive association between age (being older) and male circumcision. Education, wealth quintile, and place of residence were either not significantly related or differed in the direction of the relationship by country. Multivariate logistic regression showed three variables consistently associated with MC status: age (being older), religion (being Muslim) and ethnicity.

**Discussion:**

These data were collected prior to the scale-up of voluntary medical male circumcision (VMMC) programs in 11 of the 12 countries. As the VMMC scale-up intensifies in countries across Eastern and Southern Africa, the correlates of VMMC are likely to change, with (younger) age and education emerging as key correlates of VMMC performed in medical settings. The centuries-long tradition among Muslims to circumcise should continue to favor MC among this group. Non-circumcising ethnicities may become more open to MC if promoted as a health practice for decreasing HIV risk.

## Introduction

Voluntary medical male circumcision (VMMC) has emerged as one of the most effective means of preventing HIV transmission in countries of Eastern and Southern Africa [1]. Based on the results of three clinical trials demonstrating the efficacy of male circumcision in reducing HIV transmission (by approximately 60%), the World Health Organization and the Joint United National Programme on HIV/AIDS (UNAIDS) issued recommendations in 2007 that countries should include medical MC as part of HIV prevention interventions and that implementation should be prioritized to areas with low MC and high HIV prevalence [2]. This has led to efforts to scale-up VMMC services in 14 countries in Eastern and Southern Africa in an effort to control the HIV epidemic in these countries: Botswana, Ethiopia (Gambella National Regional State), Kenya, Lesotho, Malawi, Mozambique, Namibia, Rwanda, South Africa, Swaziland, Tanzania, Uganda, Zambia, and Zimbabwe.

The hypothesis that male circumcision (MC) might protect against HIV infection was first suggested in 1986 [3] and subsequently supported by ecological descriptions of areas with low prevalence of MC and high HIV prevalence in sub-Saharan Africa in the late 1980 s [1], [4], [5]. The Demographic and Health Survey (DHS) first included a question on male circumcision in its survey in Kenya in 2003, and since then it has measured MC prevalence rates in 11 other countries in Eastern and Southern Africa. MC prevalence ranges from a low of 8.2 percent in Swaziland (2006/2007) to a high of 92.2 percent in Ethiopia (2005), but varies markedly by region within some countries. For example, in Kenya the national MC prevalence rate was 85.9 percent as of 2009, in contrast to 44.3 percent in the province of Nyanza. Similarly, in Tanzania, the national MC prevalence rate was 72.3 percent, compared to 29.2 percent in Shinyang.

DHS data on MC prevalence have been collected between 2003 and 2011 (the most recent date is listed in Table 1). The scale-up of VMMC began in 2008 in both Orange Farm, South Africa [6] and Kisumu, Kenya [7] as a public health intervention, once the clinical trials demonstrated the efficacy of MC in reducing HIV transmission by approximately 60 percent. Subsequently, other countries have launched VMMC scale-up activities. Countries reporting the largest number of VMMC procedures performed to date are South Africa (303,534), Kenya (428,852), and Zambia (216,112), whereas the countries demonstrating the most progress toward the target of 80 percent MC coverage are Kenya (50%) and Ethiopia (38%) [8]. DHS data on MC prevalence predates the VMMC scale up in all countries except Kenya [9].

**Table 1:** Socio-demographic Profile of Men in 12 Eastern and Southern African Countries.

Country	Adult HIV Prev. (%)	DHS Year	Sample Size	Age Group (%)				Education (%)				Wealth (%)					Residence (%)	
				15–24	25–34	35–44	45+	None	Primary	Second	Higher	Poorest	Poorer	Middle	Richer	Richest	Urban	Rural
Ethiopia	1.3	2011	14,110	37.8	26.8	19.6	15.8	32.6	51.0	9.4	7.0	16.7	18.6	19.5	20.9	24.3	22.0	78.0
Kenya	6.1	2008/9	3,465	40.6	27.2	18.8	13.4	4.1	51.9	34.3	9.8	14.5	17.4	17.9	22.3	28.0	26.1	73.9
Lesotho	23.1	2009	3,317	44.3	25.9	14.7	15.1	12.9	49.0	32.2	5.9	14.6	19.6	21.9	21.2	22.7	28.0	72.0
Malawi	10.8	2010	7,175	41.6	28.5	18.5	11.4	6.6	63.1	27.1	3.2	14.4	19.3	20.1	20.4	25.8	20.9	79.1
Mozambique	11.1	2011	4,035	37.6	25.2	17.8	19.4	15.0	58.2	24.5	2.4	18.4	20.0	17.7	19.0	25.0	36.6	63.4
Namibia	13.3	2006/7	3,915	42.4	32.9	18.7	6.0	9.2	28.3	54.7	7.8	14.3	15.5	22.3	24.6	23.3	50.1	49.9
Rwanda	2.9	2010	6,329	41.2	27.6	14.5	16.6	12.0	68.3	17.6	2.1	14.8	17.5	20.6	22.0	25.1	15.9	84.1
Swaziland	26.5	2006/7	4,156	53.2	25.4	15.3	6.2	7.6	34.9	49.1	8.4	14.5	16.0	20.6	22.9	26.0	28.4	71.6
Tanzania	5.1	2010	2,527	41.9	27.5	22.5	8.1	9.5	67.7	21.8	1.1	15.9	17.7	19.4	22.6	24.5	27.4	72.6
Uganda	7.2	2011	2,295	38.0	29.8	20.0	12.2	4.5	60.2	26.9	8.4	16.1	18.8	18.6	22.5	24.0	19.9	80.1
Zambia	12.7	2007	6,500	38.2	29.7	18.3	13.8	4.6	46.3	41.0	8.1	18.7	14.8	18.2	22.8	25.6	43.2	56.8
Zimbabwe	14.7	2010/11	7,480	41.5	29.5	18.9	10.0	1.0	23.0	68.7	7.2	15.1	17.1	19.1	23.5	25.3	36.9	63.1

Note: Reference for adult HIV prevalence: 2013 UNAIDS Report on the global AIDS epidemic.

Despite the importance of MC prevalence to HIV prevention efforts in Eastern and Southern Africa, there has been no systematic analysis on the correlates of MC prevalence. In light of this gap, the current analysis was designed to:

1. to identify the correlates of male circumcision in all Eastern and Southern African countries with available data on this practice;
2. to determine if the correlates are similar across this set of countries; and
3. to discuss how the VMMC scale-up is likely to change the factors associated with male circumcision.

## Methods

The data for this analysis were obtained from the Demographic and Health Surveys (DHS) among a representative sample of men in 12 Eastern and Southern African countries: Ethiopia, Kenya, Lesotho, Malawi, Mozambique, Namibia, Rwanda, Swaziland, Tanzania, Uganda, Zambia, and Zimbabwe (all of the 14 prioritized countries except Botswana and South Africa). The survey datasets were downloaded from www.measuredhs.com.

For each country, data from the male questionnaire of the most recent available Standard DHS (ranging from 2006/7 – 2011) were analyzed, for male respondents ages 15 and older who were eligible to respond to the survey. The dependent variable was self-report of male circumcision status, based on the question: “Some men are circumcised. Are you circumcised?” and coded as a binary outcome. This question alone does not differentiate traditional circumcision from medical circumcision conducted in a clinical facility. Some countries collected additional information. For example, the questionnaires in Tanzania and Zimbabwe asked who performed the circumcision, with “traditional healer” as an option. Also the surveys in Zimbabwe and Uganda elaborated on the question cited above, to read: “Some men are circumcised, that is, the foreskin is completely removed from the penis. Are you circumcised?” These more detailed questions reveal valuable information, since traditional circumcision may be “partial” [12] and therefore not confer the same level of protection against HIV infection as medical circumcision performed by trained clinicians. However, because these clarifying questions on medical circumcision were not standard across countries, we only used the basic question that did appear on the questionnaire of all 12 countries.

Independent variables included the respondent's age at the time of the survey (grouped as 15–24, 25–34, 35–44, and 45+), education level (none, primary, secondary, and university), wealth quintile, residence (urban/rural), ethnicity, religion, and region. Due to differing numbers of groups for ethnicity, religion and region in each country, we included the five largest groups plus “other” on each of these three variables.

For each of the 12 countries in the analysis, a single logistic regression model was created in STATA 11.1, using male circumcision status as the dependent variable. Explanatory variables included four socio-demographic factors (age, education, wealth, and residence) common across every country, as well as country-specific categories for ethnicity, religion, and region. For each country, the numerically largest group on each variable was used as the reference group. Data on ethnicity were available for six countries, on religion for 11 countries, and on region for 12 countries.

## Results

The socio-demographic profile of male respondents from the DHS ages 15 and above in 12 Eastern and Southern African countries is displayed in Table 1. Consistent with demographic trends across this region, the modal group in most countries was between the ages of 15–24, with the majority of respondents living in rural areas. Education levels varied across countries, with most men having completed at least primary school. Table 1 (column 2) reflects the wide range of HIV prevalence in these countries, from 1.3% in Ethiopia to 26.5% in Swaziland.

**Figure 1:**
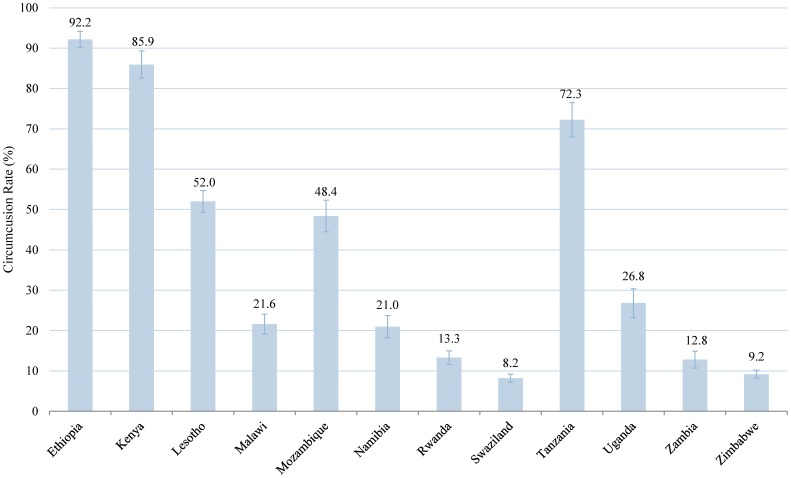
Male Circumcision Rates in Eastern and Southern African Countries. Percent circumcised among males in 12 Eastern and Southern African countries. We calculated these rates based on DHS survey data from the most recent years available. Error bars represent 95% CI.

Figure 1 shows the percent of men circumcised in each country. Total male circumcision rates ranged from 8.2 percent in Swaziland to 92.2 percent in Ethiopia. Three out of 12 countries in the study had male circumcision rates over 70 percent (Ethiopia, Kenya, and Tanzania), while in Lesotho and Mozambique about half of the male population was circumcised. By contrast, Swaziland and Zimbabwe had male circumcision rates of less than ten percent.

### Bivariate Analysis

**Figure 2:**
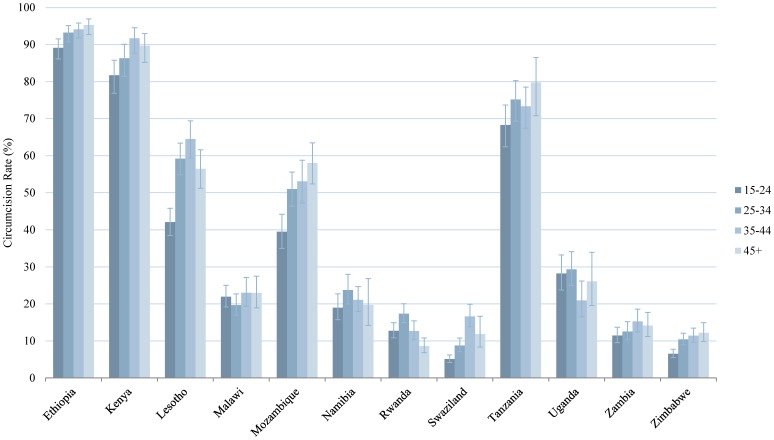
Male Circumcision Rates in Eastern and Southern African Countries, by Age. Percent circumcised among males of different age groups: 15–24, 25–34, 35–44, and 45+. Error bars represent 95% CI.

**Figure 3:**
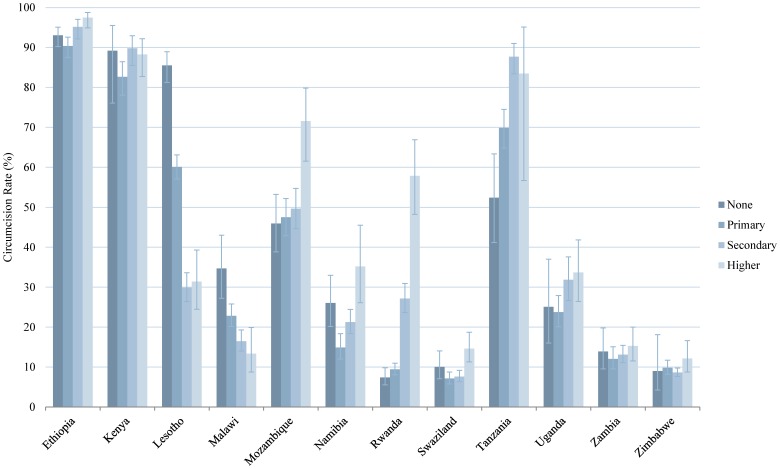
Male Circumcision Rates in Eastern and Southern African Countries, by Education. Percent circumcised among males of different educational levels: none, primary, secondary, and higher. Error bars represent 95% CI.

**Figure 4:**
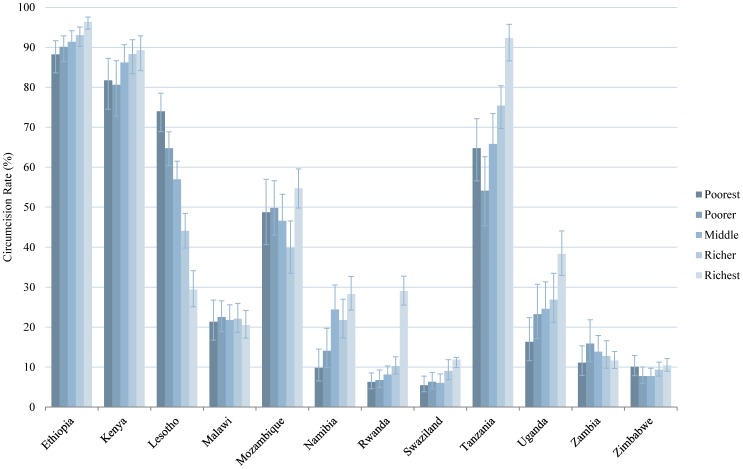
Male Circumcision Rates in Eastern and Southern African Countries, by Wealth. Percent circumcised among males of different wealth quintiles: poorest, poorer, middle, richer, and richest. Error bars represent 95% CI.

**Figure 5:**
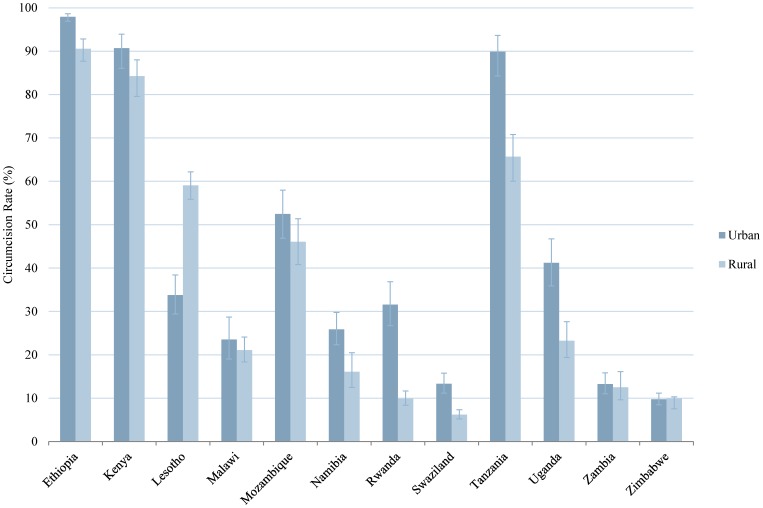
Male Circumcision Rates in Eastern and Southern African Countries, by Residence. Percent circumcised among males by place of residence: urban or rural. Error bars represent 95% CI.

In this analysis we tested the bivariate relationship between the prevalence of male circumcision and seven independent variables. The findings for four of the variables (age group, education, wealth, and residence) are presented in graphical form in Figures 2–5 and numerically in Table S1. With regard to age, in the majority of countries, male circumcision rates tracked with age group: a greater proportion of men ages 35–44 and 45+ were circumcised compared to men ages 15–24 and 25–34. In Namibia, Rwanda, and Uganda, however, men ages 25–34 had the highest rates of circumcision (Figure 2). Regarding education, in 10 of 12 countries, men with secondary or higher education were more likely to be circumcised than men with only primary or no education. The trend was reversed in Lesotho and Malawi, in which those men with no education were circumcised at the highest rates and by a sizable margin (Figure 3). Wealth quintile showed a similar pattern in the bivariate analysis in 9 of 12 countries, with those men in the richest quintile more likely to be circumcised, except in Lesotho, Malawi, and Zambia (Figure 4). In 10 of 12 countries, a greater percentage of men in urban areas were circumcised compared to those living in rural areas. However, the reverse was true in Zimbabwe and particularly Lesotho, where a much greater percentage of men in rural areas were circumcised (Figure 5).

Three final variables tested in the bivariate analysis were ethnicity, religion, and region. These results are presented in Table S2. Circumcision varied widely among the different ethnic groups. In Ethiopia and Kenya, four of the top five ethnic groups had circumcision rates greater than 94%, whereas one ethnic group in each country (Sidama and Luo, respectively) had a markedly lower circumcision rate, pointing to cultural factors influencing circumcision status. Similarly, in Malawi, the Yao ethnic group had a male circumcision rate of 87.2%, four times the national circumcision rate of 21.6%. With regards to religion, male circumcision tends to be far more prevalent among Muslims than among members of other religious groups. In Ethiopia, Kenya, Malawi, and Uganda, circumcision rates are greater than 90% among Muslims. Circumcision is also frequently practiced among Muslims in Mozambique (85.2%), Rwanda (72.6%), and Zambia (62.5%). In all seven countries where Muslims were among the top five largest religious groups, the circumcision rate among Muslims in each country was greater than the national circumcision rate, on average by a factor of three. In Rwanda, for example, the prevalence of male circumcision among Muslims was more than five times the national prevalence. Other religious groups, with minor variations, tended to track more closely with the national rates. Regarding region, there were stark differences by country, which are of country-specific interest but less so for a cross-national analysis. The urban centers of Addis Ababa, Ethiopia, and Dar es Salaam, Tanzania, had circumcision rates above 95%, while Lusaka, Zambia, and Harare, Zimbabwe, had circumcision rates around ten percent. In Mozambique, the five largest regions in terms of population size included Zambezia (49.1% circumcised), Nampula (84.0%), Tete (2.0%), Cabo Delgado (72.2%), and Sofala (16.3%), suggesting a wide range of circumcision rates regionally.

Circumcision rates and relative size of each religious group, ethnic group, and region are presented in Table S2.

### Multivariate Analysis

Table 2 presents the results of a logistic regression analysis with “male circumcision status” as the dependent variable and the seven variables described above (age, education, wealth, residence, religion, ethnicity and region) as independent variables. The analysis was run separately for each of the 12 countries. Reference groups were defined as “ages 15–24”, “no education”, “poorest wealth quintile”, “rural”, as well as the largest ethnic group, religious group, and region by population. Odds ratios for being circumcised are displayed in Table 2, with significant p-values indicated by asterisks.

**Table 2:** Odds Ratios related to Male Circumcision in 12 Eastern and Southern African countries: Age, Education, Wealth, and Residence.

Country	Age Group (reference group = ages 15–24)			Education (reference group = no education)			Wealth (reference group = poorest quintile)				Residence (ref = rural)
	25–34	35–44	45+	Primary	Secondary	Higher	Poorer	Middle	Richer	Richest	Urban
Ethiopia	2.18***	2.70***	3.40***	1.10	1.45	2.10	1.12	1.33	1.52	1.33	2.85*
Kenya	1.98*	4.32***	2.61***	0.89	2.43	2.09	1.41	1.86*	2.53*	2.73*	0.67
Lesotho	2.00***	2.38***	1.17	0.30***	0.11***	0.14***	0.94	0.88	0.67*	0.53**	0.79
Malawi	0.96	1.15	1.15	0.97	0.71	0.63	1.14	1.04	1.00	1.00	1.07
Mozambique	1.74***	1.95***	2.76***	1.48*	1.77**	3.57***	0.86	0.93	0.80	1.47	1.02
Namibia	1.13	1.03	0.85	0.52***	0.60*	0.99	1.73*	3.06***	2.54**	3.38***	1.20
Rwanda	1.53***	1.10	0.79	1.14	3.02***	7.02***	1.01	1.24	1.48*	2.93***	1.99***
Swaziland	1.54**	3.23***	2.13**	0.94	0.81	0.83	1.20	1.04	1.32	1.37	1.79**
Tanzania	1.73***	1.70**	2.23**	1.59*	4.36***	0.62	0.63*	0.89	1.11	3.27**	1.51
Uganda	1.27	0.84	1.01	1.08	1.55	1.61	1.33	1.48	1.55	2.18*	2.02**
Zambia	1.07	1.25*	1.08	0.80	0.97	1.26	1.06	0.86	0.76	0.55*	1.46
Zimbabwe	1.66***	1.78***	1.87***	1.16	1.00	1.09	0.80	0.78	0.92	1.02	1.11

Note: Statistical significance within the logistic regression model has been marked by asterisks: * p<0.05, ** p<0.01, and *** p<0.001.

Three variables emerged as significant predictors of male circumcision in multiple countries: age, religion, and ethnicity. In most countries, age was a significant predictor of circumcision status, with those men older than 24 more likely to be circumcised than men ages 15–24, tracking well with the results of the bivariate analysis. The size of the effect tended to be greater in the age groups 35–44 and 45+ (up to two or three times more likely to be circumcised than a male ages 15–24), but this was not always the case. In Malawi, Namibia, and Uganda, age was not a statistically significant factor.

Ethnicity was found to be statistically correlated with male circumcision in all six countries for which data on ethnic groups were available. In these countries, at least one ethnic group among the top five was a significant predictor of male circumcision, when compared against the reference group (the largest ethnic group in the country by population). In Kenya, for example, membership in the Luo group is a statistically significant negative predictor of male circumcision (p<0.01), whereas in Malawi, a male in the Yao group is ten times more likely to be circumcised than those in the Chewa group (p<0.001).

With respect to religion, Muslims were far more likely to be circumcised than men of other religions in nearly all countries where Muslims were listed as a separate religious group. For example, in Uganda, a Muslim male is about one hundred times more likely than a Catholic male to be circumcised. Statistically significant odds ratios for Muslims being circumcised, compared to the reference groups, were calculated in Malawi, Mozambique, Rwanda, Uganda, and Zambia.

Education, wealth, and residence variables were not statistically significant predictors of male circumcision status in many of the countries in the study. Where these factors were significant, there was no clear trend in terms of directionality of the effect. For example, in Lesotho and Namibia, education level was a negative predictor of male circumcision, while in Mozambique, Rwanda, and Tanzania, the opposite was true: education level was a positive predictor of male circumcision. In Kenya, Namibia, and Rwanda, those men in higher wealth quintiles were significantly more likely to be circumcised, however in Lesotho there is a significant negative association between wealth quintile and circumcision status. Living in an urban area versus a rural area was a statistically-significant positive predictor of male circumcision in only four countries: Ethiopia, Rwanda, Swaziland, and Uganda.

In sum, the multivariate analysis of factors associated with MC status in these 12 countries showed age, religion, and ethnicity to be the strongest correlates of this practice. MC prevalence was higher among men 25 years and older, among Muslim men, and among specific ethnicities that varied by country. The effects of education, wealth, or urban residence – found in the bivariate analysis – did not emerge in the multivariate analysis or differed in the direction of the effect.

## Limitations

The authors recognize several limitations of this analysis. First, DHS data on MC prevalence were not available for two of the 14 countries prioritized for the VMMC scale up: Botswana and South Africa. Given the large population of South Africa (52 million) and high HIV prevalence (17.9%), the lack of accessible survey data on HIV and MC from South Africa is regrettable. Second, although a number of countries have collected MC prevalence data in previous rounds of the DHS, this analysis only examines the latest DHS surveys with publicly-available datasets. Third, the data on MC status were self-reported and thus subject to social desirability bias, if the respondent felt that one answer would be viewed more favorably than another. However, in many African societies, male circumcision (or lack of it) is a stronger marker of cultural identity. For men whose status conformed to the cultural marker, there would be little reason not to give an accurate reply regarding their own circumcision status.

## Discussion

The timing of data collection – prior to the launch of the VMMC scale-up in 11 of these 12 countries – has important implications for the findings. Specifically, certain factors that were strongly associated with MC prevalence prior to the launch may no longer hold in the future, as the rollout of VMMC services expands through this region. For example, in 9 of the 12 countries, MC prevalence increased with age: it was higher among men 34–44 and 45+ years old compared to younger men. Yet in country after country in Eastern and Southern Africa, VMMC programs are finding it far easier to attract adolescent boys and young men under 25 years of age than men over 25 [10], [11]. Data from Orange Farm, South Africa (site of the first of the three clinical trials on male circumcision, where the intervention was widely scaled up starting in 2008) illustrates how this relationship changed in the presence of active VMMC programming. Prior to the scale-up, the percent of men 15–49 who were circumcised increased with age (consistent with the findings in this analysis). However, after a period of only three years, the opposite was observed: the percent circumcised decreased monotonically with each five year age grouping [6]. This same trend is likely to occur in other areas where the VMMC scale-up reaches large segments of the male population, with greater uptake among men under 25 years of age.

In the current analysis, education, wealth quintile, and urban/rural status either showed no relationship to circumcision status or were related but in opposite directions in different countries (i.e., failed to show a consistent direction of influence across countries). This lack of an effect with consistent directionality contrasts sharply to the strong influence that these three variables have on the majority of behaviors promoted by public health interventions (e.g., contraceptive use, condom use for HIV prevention, delivery with a skilled birth attendant) [12], [13]. The likely explanation again relates to the fact that these data predate the launch of VMMC programs. Many of the male respondents in these DHS surveys would have undergone traditional circumcision, not affected by education or place of residence [14]. However, as male circumcision becomes a public health intervention, these factors are likely to come into play. For example, in Orange Farm, in the post-intervention survey, circumcised males were more likely to be in school and to be educated than their uncircumcised counterparts [6]. Also in Tanzania, during a high –volume VMMC campaign, differences in MC were attributed to place of residence” with urban men having a greater prevalence of MC than their counterparts [11].

Religion and ethnicity – strong correlates of VMMC prior to the scale-up – are likely to remain predictors of VMMC, even with the VMMC scale-up in many countries. The finding from the current analysis showing markedly higher MC prevalence among Muslim men confirmed the claims made elsewhere that male circumcision is practiced for religious, social, and medical reasons [1]. However, unlike other public health topics where religious doctrine dictates against a health practice (e.g., the Roman Catholic Church and the use of modern contraception), there is no religious doctrine that prohibits male circumcision. One observed change with the VMMC scale-up found in other research is that many families that practiced male circumcision for cultural or religious reasons still seek out the procedure for their adolescent sons, but opt for medical rather than traditional circumcision [15]–[17].

Ethnicity is a powerful factor because male circumcision defines cultural identity among certain African tribes, as documented in other studies [18], [19]. For some, male circumcision is considered to be a mark of citizenship, a religious or cultural affiliation; for others it is a sign of “otherness” that signals exclusion, marginalization or oppression [20]. Circumcision is tightly linked to masculinity and sexuality. Ethnic groups that do not traditionally practice circumcision often disapprove of it, using derogatory terms in referring to a circumcised man. By contrast, other studies have shown that young men from groups practicing circumcision fear stigmatization if they do not go through with the procedure [21].

Although initially considered a significant barrier to male circumcision, findings from other research suggest that ethnicity and culture may not be insurmountable obstacles. In the case of Kenya, the national program targeted the province of Nyanza in its early programming, precisely because of the low rates of circumcision among the Luo population inhabiting this region. With culturally sensitive programming that involved traditional leaders and strong community participation, this program has been among the most successful to date in terms of circumcisions performed [7]. One recommendation from formative research conducted in Turkana County, Kenya (another area of relatively low MC prevalence) was to promote VMMC as a medical intervention valuable for reducing HIV transmission, without directly evoking the aspect of cultural identity related to circumcision. Those researchers found that stigma associated with getting circumcised (going against cultural norms) was balanced by the stigma of not being modern or of risking HIV. Elders were able to accept the notion of male circumcision for its preventive health benefits, independent of cultural practice [22]. Khumalo-Sakutukwa described similar findings among non-circumcising Zulus from the community of Vulindlela [18]. The negative characterizations of circumcised men when discussed in the context of cultural identity became more positive when the discussion turned to the benefits in terms of hygiene and disease prevention.

In sum, this analysis yields a greater understanding of the factors that influenced the practice of male circumcision prior to the VMMC scale-up, when the primary drivers were age (being older), religion (being Muslim), and ethnicity. The results from other studies and program reports show far greater demand for VMMC among men under 25 than older men; thus, it would be misleading to expect male circumcision prevalence to continue to be associated with higher age. As programs reach saturation (80% prevalence among the male population), the age effect is likely to diminish. By contrast, the associations of religion and ethnicity with male circumcision have strong implications for demand creation. Muslim populations have a strong disposition toward VMMC, which serves as an enabling factor. By comparison, ethnicity can serve as an enabler or an impediment, depending on group. Non-circumcising tribes will require particular education and motivation to overcome existing cultural norms against male circumcision; yet these may also be the most important populations to prioritize. For example, the Kenya program focused its most intensive efforts on Nyanza Province, precisely because of the strong tradition among members of the Luo tribe *not* to circumcise.

As the VMMC scale-up intensifies in countries across Eastern and Southern Africa, the correlates of VMMC are likely to change, with (younger) age and education emerging as key correlates of VMMC performed in medical settings. There is no reason to believe that Muslims will move away from the practice. However, the classification of ethnic groups as circumcising versus non-circumcising may become less clear-cut, especially if VMMC programs are able to approach local communities with programming that emphasizes the health benefits of the practice.

## Supporting Information

Table S1 **Bivariate Analysis of Male Circumcision in Eastern and Southern African Countries.** Circumcision rates (%) among males in 12 Eastern and Southern African countries, grouped by age, education, wealth, and residence. We calculated these rates based on DHS survey data from the most recent years available.
(DOCX)

Table S2 **Circumcision Rates (%) and Odds Ratios among Ethnic Groups, Religions, and Regions in Eastern and Southern African Countries.** Circumcision rates (%) are shown in Table S2 for the major ethnic groups, religions, and regions in 12 Eastern and Southern African countries. They represent the five largest ethnicities, religions, and regions by population size. The remaining populations are consolidated into the “Other” category. Also displayed are the relative size of each group (% of total population) and the odds ratio of being circumcised according to the logistic regression model. The largest ethnic group, religion, and region in each country were selected as reference groups.
(DOCX)
