# Supplementary material for: Correlates of Male Circumcision in Eastern and Southern African Countries: Establishing a Baseline Prior to VMMC Scale-Up
Source: PLoS One. 2014 Jun 23;9(6):e100775. doi: 10.1371/journal.pone.0100775 (PMC4067410; doi:10.1371/journal.pone.0100775)
Supplement: Table S1 — Bivariate Analysis of Male Circumcision in Eastern and Southern African Countries. Circumcision rates (%) among males in 12 Eastern and Southern African countries, grouped by age, education, wealth, and residence. We calculated these rates based on DHS survey data from the most recent years available. (DOCX) [file pone.0100775.s001.docx]

**Table S1**

**Bivariate Analysis of Male Circumcision in Eastern and Southern African Countries, Circumcision Rates (%)**

| **Country** | **Total Population** | **Age Group** | | | | **Education** | | | | **Wealth** | | | | | **Residence** | |
| --- | --- | --- | --- | --- | --- | --- | --- | --- | --- | --- | --- | --- | --- | --- | --- | --- |
|  |  | 15-24 | 25-34 | 35-44 | 45+ | None | Primary | Second. | Higher | Poorest | Poorer | Middle | Richer | Richest | Urban | Rural |
| Ethiopia | 92.2 | 89.1 | 93.3 | 94.1 | 95.3 | 93.1 | 90.4 | 95.2 | 97.5 | 88.2 | 90.1 | 91.4 | 93.1 | 96.4 | 97.9 | 90.6 |
| Kenya | 85.9 | 81.7 | 86.3 | 91.7 | 89.7 | 89.2 | 82.7 | 89.8 | 88.3 | 81.7 | 80.7 | 86.2 | 88.3 | 89.3 | 90.7 | 84.2 |
| Lesotho | 52.0 | 42.1 | 59.2 | 64.5 | 56.5 | 85.5 | 60.1 | 29.9 | 31.4 | 74.0 | 64.8 | 57.0 | 44.1 | 29.4 | 33.8 | 59.1 |
| Malawi | 21.6 | 21.9 | 19.7 | 23.0 | 22.9 | 34.7 | 22.8 | 16.5 | 13.4 | 21.3 | 22.5 | 21.8 | 22.1 | 20.5 | 23.5 | 21.1 |
| Mozambique | 48.4 | 39.5 | 51.0 | 53.1 | 58.0 | 45.9 | 47.5 | 49.7 | 71.6 | 48.8 | 49.8 | 46.6 | 39.9 | 54.7 | 52.4 | 46.1 |
| Namibia | 21.0 | 19.0 | 23.7 | 21.1 | 19.8 | 26.1 | 14.9 | 21.3 | 35.2 | 9.8 | 14.1 | 24.4 | 21.8 | 28.3 | 25.9 | 16.1 |
| Rwanda | 13.3 | 12.7 | 17.4 | 12.6 | 8.6 | 7.4 | 9.4 | 27.1 | 57.9 | 6.3 | 6.8 | 8.1 | 10.2 | 29.0 | 31.6 | 9.9 |
| Swaziland | 8.2 | 5.1 | 8.8 | 16.6 | 11.9 | 10.0 | 7.1 | 7.6 | 14.6 | 5.5 | 6.3 | 6.1 | 9.0 | 11.9 | 13.3 | 6.2 |
| Tanzania | 72.3 | 68.3 | 75.2 | 73.3 | 79.8 | 52.4 | 69.9 | 87.7 | 83.5 | 64.8 | 54.1 | 65.8 | 75.4 | 92.4 | 89.9 | 65.7 |
| Uganda | 26.8 | 28.2 | 29.3 | 20.9 | 26.1 | 25.1 | 23.8 | 31.9 | 33.7 | 16.3 | 23.3 | 24.6 | 26.9 | 38.4 | 41.2 | 23.3 |
| Zambia | 12.8 | 11.4 | 12.5 | 15.3 | 14.1 | 13.9 | 12.0 | 13.1 | 15.3 | 11.1 | 15.9 | 13.9 | 12.8 | 11.6 | 13.3 | 12.5 |
| Zimbabwe | 9.2 | 6.5 | 10.4 | 11.4 | 12.2 | 9.0 | 9.8 | 8.6 | 12.2 | 10.1 | 7.7 | 7.8 | 9.4 | 10.4 | 9.7 | 9.9 |
