# Supplementary material for: Correlates of Male Circumcision in Eastern and Southern African Countries: Establishing a Baseline Prior to VMMC Scale-Up
Source: PLoS One. 2014 Jun 23;9(6):e100775. doi: 10.1371/journal.pone.0100775 (PMC4067410; doi:10.1371/journal.pone.0100775)
Supplement: Table S2 — Circumcision Rates (%) and Odds Ratios among Ethnic Groups, Religions, and Regions in Eastern and Southern African Countries. Circumcision rates (%) are shown in Table S2 for the major ethnic groups, religions, and regions in 12 Eastern and Southern African countries. They represent the five largest ethnicities, religions, and regions by population size. The remaining populations are consolidated into the “Other” category. Also displayed are the relative size of each group (% of total population) and the odds ratio of being circumcised according to the logistic regression model. The largest ethnic group, religion, and region in each country were selected as reference groups. (DOCX) [file pone.0100775.s002.docx]

**Table S2**

**Circumcision Rates (%) and Odds Ratios among Ethnic Groups, Religions, and Regions in Eastern and Southern African Countries**

| **Country** | **Ethnic Group** | | | | **Religion** | | | | **Region** | | | |
| --- | --- | --- | --- | --- | --- | --- | --- | --- | --- | --- | --- | --- |
|  | Name | Percent of Total Population | Circumcision Rate (%) | Odds Ratio | Name | Percent of Total Population | Circumcision Rate (%) | Odds Ratio | Name | Percent of Total Population | Circumcision Rate (%) | Odds Ratio |
| Ethiopia | Oromo | 35.4 | 95.2 | 1.00 | Orthodox | 48.6 | 96.5 | 1.00 | Oromiya | 38.2 | 93.9 | 1.00 |
|  | Amhara | 32.1 | 96.0 | 0.46 | Muslim | 29.2 | 95.8 | 0.76 | Amhara | 27.7 | 95.6 | 1.28 |
|  | Tigrie | 6.5 | 97.0 | 0.74 | Protestant | 18.8 | 79.4 | 0.16*** | S.N.N.P. | 17.9 | 78.9 | 1.65 |
|  | Sidama | 3.8 | 66.2 | 0.19* | Catholic | 1.0 | 84.5 | 0.24** | Tigray | 6.1 | 96.5 | 0.98 |
|  | Welaita | 2.9 | 97.2 | 2.23 | Traditional | 0.7 | 77.7 | 0.17*** | Addis Ababa | 5.1 | 99.1 | 3.48* |
|  | Other | 19.4 | 83.0 | 0.19** | Other | 1.8 | 60.2 | 0.09*** | Other | 4.9 | 95.4 | 2.93 |
| Kenya | Kikuyu | 17.7 | 98.2 | 1.00 | Protestant | 62.9 | 85.1 | 1.00 | Rift Valley | 27.0 | 91.1 | 1.00 |
|  | Luhya | 17.4 | 96.1 | 0.44 | Catholic | 25.6 | 84.6 | 0.65* | Eastern | 16.8 | 96.6 | 1.87 |
|  | Kalenjin | 13.3 | 94.2 | 0.62 | Muslim | 6.4 | 96.5 | 1.64 | Nyanza | 15.9 | 44.3 | 1.12 |
|  | Luo | 13.0 | 21.0 | 0.004*** | No Religion | 4.4 | 92.9 | 0.88 | Western | 10.7 | 93.7 | 2.04 |
|  | Kamba | 11.6 | 99.2 | 2.48 | Other | 0.7 | 71.5 | 0.22 | Central | 10.6 | 97.0 | 1.24 |
|  | Other | 27.0 | 92.9 | 0.24** | - | - | - | - | Other | 19.0 | 93.4 | 3.60* |
| Lesotho | - | - | - | - | Catholic | 42.5 | 50.3 | 1.00 | Maseru | 25.5 | 35.5 | 1.00 |
|  | - | - | - | - | Evangelical | 19.9 | 47.6 | 1.02 | Leribe | 16.0 | 57.1 | 2.52*** |
|  | - | - | - | - | Pentecostal | 18.7 | 59.6 | 1.27* | Berea | 15.2 | 47.9 | 1.72** |
|  | - | - | - | - | Anglican | 8.9 | 48.9 | 0.85 | Mafeteng | 9.9 | 53.3 | 1.97*** |
|  | - | - | - | - | No Religion | 5.1 | 62.1 | 0.94 | Mohale's Hoek | 8.6 | 60.2 | 1.74** |
|  | - | - | - | - | Other | 4.9 | 50.8 | 1.19 | Other | 24.9 | 64.7 | 2.31*** |
| Malawi | Chewa | 33.3 | 6.0 | 1.00 | Christian,oth | 35.2 | 13.4 | 1.00 | Central | 45.2 | 10.1 | 1.00 |
|  | Lomwe | 17.9 | 29.9 | 3.65*** | Catholic | 22.3 | 8.9 | 0.76 | Southern | 43.8 | 38.3 | 3.40*** |
|  | Yao | 13.1 | 87.2 | 10.84*** | CCAP | 16.7 | 8.6 | 0.91 | Northern | 11.0 | 2.6 | 0.67 |
|  | Ngoni | 12.8 | 5.8 | 0.82 | Muslim | 12.2 | 94.1 | 48.21*** | - | - | - | - |
|  | Tumbuka | 8.5 | 1.0 | 0.29** | SDA | 7.1 | 18.2 | 1.35 | - | - | - | - |
|  | Other | 14.3 | 13.9 | 1.54 | Other | 6.6 | 11.3 | 1.17 | - | - | - | - |
| Mozambique | Emakhuwa | 26.9 | 79.5 | 1.00 | Catholic | 31.1 | 55.1 | 1.00 | Zambezia | 18.6 | 49.1 | 1.00 |
|  | Xichanga | 10.0 | 28.3 | 0.25*** | Islamic | 19.5 | 85.2 | 3.06*** | Nampula | 15.6 | 84.0 | 3.45** |
|  | Cisena | 9.8 | 13.6 | 0.38* | No Religion | 17.4 | 24.0 | 0.42*** | Tete | 12.2 | 2.0 | 0.07*** |
|  | Cicewa | 8.8 | 4.7 | 0.18** | Evangelical | 13.4 | 32.3 | 0.51*** | Cabo Delgado | 9.9 | 72.2 | 1.83 |
|  | Elomwe | 8.2 | 64.9 | 2.34* | Zion/Sião | 11.5 | 27.8 | 0.53*** | Sofala | 9.9 | 16.3 | 0.36** |
|  | Other | 36.4 | 47.1 | 0.75 | Other | 7.1 | 41.3 | 0.70* | Other | 33.7 | 50.8 | 1.96** |
| Namibia | - | - | - | - | Protestant | 70.6 | 21.2 | 1.00 | Khomas | 25.1 | 26.6 | 1.00 |
|  | - | - | - | - | Catholic | 26.4 | 18.5 | 0.76* | Erongo | 9.3 | 18.7 | 0.72 |
|  | - | - | - | - | No Religion | 2.4 | 38.1 | 1.74 | Kavango | 8.4 | 30.5 | 2.70** |
|  | - | - | - | - | Other | 0.6 | 42.9 | 1.87 | Oshikoto | 8.2 | 8.3 | 0.46* |
|  | - | - | - | - | - | - | - | - | Omusati | 8.2 | 7.9 | 0.51 |
|  | - | - | - | - | - | - | - | - | Other | 40.8 | 21.2 | 1.19 |
| Rwanda | - | - | - | - | Catholic | 48.5 | 9.7 | 1.00 | East | 25.2 | 9.7 | 1 |
|  | - | - | - | - | Protestant | 35.2 | 14.9 | 1.43*** | West | 23.5 | 20.4 | 3.29*** |
|  | - | - | - | - | Adventist | 11.8 | 13.7 | 1.49* | South | 22.8 | 4.5 | 0.50*** |
|  | - | - | - | - | Muslim | 1.9 | 72.6 | 29.92*** | North | 16.0 | 5.2 | 0.59** |
|  | - | - | - | - | No religion | 1.7 | 11.2 | 0.97 | Kigali City | 12.5 | 33.9 | 1.29 |
|  | - | - | - | - | Other | 0.9 | 20.4 | 1.44 | - | - | - | - |
| Swaziland | - | - | - | - | Zionist | 37.1 | 6.5 | 1.00 | Manzini | 32.5 | 9.2 | 1.00 |
|  | - | - | - | - | No Religion | 18.7 | 8.9 | 1.23 | Hhohho | 26.5 | 9.2 | 0.94 |
|  | - | - | - | - | Protestant | 17.8 | 8.0 | 1.07 | Lubombo | 20.8 | 6.9 | 0.71* |
|  | - | - | - | - | Charismatic | 10.8 | 10.2 | 1.36 | Shiselwe | 20.3 | 6.7 | 0.96 |
|  | - | - | - | - | Apostolic Sect | 6.1 | 9.1 | 1.30 | - | - | - | - |
|  | - | - | - | - | Other | 9.6 | 11.1 | 1.43 | - | - | - | - |
| Tanzania | - | - | - | - | - | - | - | - | Mwanza | 10.9 | 49.4 | 1.00 |
|  | - | - | - | - | - | - | - | - | Dar es Salaam | 8.2 | 95.5 | 7.56** |
|  | - | - | - | - | - | - | - | - | Shinyang | 6.7 | 29.2 | 0.40* |
|  | - | - | - | - | - | - | - | - | Kagera | 6.4 | 38.2 | 0.74 |
|  | - | - | - | - | - | - | - | - | Morogoro | 5.8 | 96.1 | 25.30*** |
|  | - | - | - | - | - | - | - | - | Other | 62.0 | 79.2 | 4.24*** |
| Uganda | Baganda | 16.7 | 31.1 | 1.00 | Catholic | 44.0 | 14.8 | 1.00 | Western | 14.3 | 29.1 | 1.00 |
|  | Banyankole | 10.0 | 18.2 | 1.36 | Protestant | 31.8 | 19.2 | 1.09 | Eastern | 13.1 | 36.8 | 3.07* |
|  | Basoga | 9.0 | 50.5 | 2.03 | Muslim | 12.6 | 94.0 | 103.81*** | Southwest | 12.7 | 8.8 | 0.23** |
|  | Bakiga | 7.4 | 10.3 | 0.83 | Pentecostal | 8.3 | 23.1 | 1.35 | Central 2 | 11.0 | 25.3 | 0.33* |
|  | Iteso | 6.7 | 7.7 | 0.13*** | SDA | 1.8 | 21.4 | 1.00 | East Central | 10.9 | 43.6 | 0.63 |
|  | Other | 50.2 | 27.9 | 1.59 | Other | 1.5 | 3.5 | 0.1237* | Other | 38.0 | 24.2 | 0.30** |
| Zambia | Bemba | 20.7 | 6.6 | 1.00 | Protestant | 74.6 | 13.2 | 1.00 | Copperbelt | 19.1 | 14.7 | 1.00 |
|  | Tonga | 11.0 | 2.6 | 0.31** | Catholic | 21.9 | 9.4 | 0.90 | Lusaka | 17.7 | 10.3 | 0.71 |
|  | Chewa | 8.3 | 4.8 | 1.20 | Muslim | 0.5 | 62.5 | 25.48*** | Northern | 13.4 | 3.2 | 0.20*** |
|  | Nsenga | 5.5 | 3.7 | 0.71 | Other | 3.0 | 19.6 | 1.48 | Eastern | 13.4 | 3.1 | 0.16*** |
|  | Lozi | 5.4 | 8.1 | 0.66 | - | - | - | - | Southern | 10.2 | 4.3 | 0.68 |
|  | Other | 49.1 | 20.7 | 2.72*** | - | - | - | - | Other | 26.2 | 26.4 | 1.94** |
| Zimbabwe | - | - | - | - | Apostolic | 27.3 | 8.2 | 1.00 | Harare | 18.3 | 8.6 | 1.00 |
|  | - | - | - | - | No Religion | 21.6 | 8.0 | 0.94 | Manicaland | 13.6 | 13.1 | 1.92** |
|  | - | - | - | - | Pentecostal | 14.3 | 9.5 | 1.10 | Midlands | 12.5 | 9.8 | 1.32 |
|  | - | - | - | - | Protestant | 14.0 | 9.5 | 1.10 | MashonaWest | 12.3 | 7.6 | 1.05 |
|  | - | - | - | - | Catholic | 10.3 | 9.4 | 1.06 | MashonaCentral | 10.3 | 5.8 | 0.82 |
|  | - |  | - | - | Other | 12.6 | 12.3 | 1.42* | Other | 33.0 | 9.3 | 1.25 |

*Note: Statistical significance within the logistic regression model has been marked by asterisks: * p<0.05, ** p<0.01, and *** p<0.001*
